# Lipotoxicity suppresses the synthesis of growth hormone in pituitary somatotrophs via endoplasmic reticulum stress

**DOI:** 10.1111/jcmm.16532

**Published:** 2021-05-04

**Authors:** Ying Gong, Jianmei Yang, Shuoshuo Wei, Rui Yang, Ling Gao, Shanshan Shao, Jiajun Zhao

**Affiliations:** ^1^ Department of Endocrinology Shandong Provincial Hospital Cheeloo College of Medicine Shandong University Jinan China; ^2^ Shandong Provincial Key Laboratory of Endocrinology and Lipid Metabolism Jinan China; ^3^ Shandong Institute of Endocrine and Metabolic Disease Jinan China; ^4^ Department of Pediatric Endocrinology Shandong Provincial Hospital Affiliated to Shandong First Medical University Jinan China; ^5^ Experimental Animal Center Shandong Provincial Hospital Affiliated to Shandong First Medical University Jinan China; ^6^ Department of Endocrinology Shandong Provincial Hospital Affiliated to Shandong First Medical University Jinan China

**Keywords:** anterior pituitary, endoplasmic reticulum stress, growth hormone, high‐fat diet, lipotoxicity, Pit‐1

## Abstract

Lipotoxicity has been shown to cause dysfunction of many organs and tissues. However, it is unclear whether lipotoxicity is harmful to the somatotrophs, a kind of cell that synthesize growth hormone (GH) in the pituitary. In this study, we performed an epidemiological study, serum levels of triglyceride (TG) and GH showed a negative correlation, even after adjustment for potential confounders. In an animal study, male Sprague‐Dawley rats were fed a high‐fat diet (HFD) or a control diet for 28 weeks. HFD rats showed impaired GH synthesis, resulting in a decrease in circulating GH levels. The expression of pituitary Pit‐1, a key transcription factor of GH, was inhibited. We found that the inositol‐requiring enzyme 1α (IRE1α) pathway of endoplasmic reticulum (ER) stress was triggered in HFD rat pituitary glands and palmitic acid‐treated GH3 cells, respectively. On the contrary, applying 4‐phenyl butyric acid (4‐PBA) to alleviate ER stress or 4µ8c to specifically block the IRE1α pathway attenuated the impairment of both Pit‐1 and GH expression. In conclusion, we demonstrated that lipotoxicity directly inhibits the synthesis of GH, probably by reducing Pit‐1 expression. The IRE1α signaling pathway of ER stress may play an important role in this process.

## 1 INTRODUCTION

High‐fat diet (HFD)‐induced hyperlipidemia is constant threats to human health worldwide and can result in the development of multiple diseases, such as type 2 diabetes, coronary heart disease, musculoskeletal diseases, and several cancers.^1^ Persistent hyperlipidemia, especially high triglyceride (TG) levels in the circulation, causes deleterious lipid accumulation in non‐adipose tissues in a process, known as lipotoxicity.^2^ In recent years, lipotoxicity has been shown to cause dysfunction in many organs and tissues.^3^, ^4^, ^5^ According to our previous study, there are significant correlations between lipotoxicity and the levels of pituitary‐thyroid axis hormones.^6^ These findings indicate that the pituitary may be a potential target organ of lipotoxicity. However, to date, it is unclear whether lipotoxicity can directly disrupt the function of the pituitary in synthesizing growth hormone (GH).

Growth hormone is a kind of pleiotropic polypeptide that is synthesized and secreted by anterior pituitary somatotrophs, and its physiological function is to promote anabolic metabolism and growth.^7^ Circulating GH level is mainly regulated by the coordinated control of two hypothalamic neuropeptides, GH‐releasing hormone (GHRH), which stimulates GH release, and somatostatin (SS), which has an inhibitory action.^8^ Many physiological and pathological factors, including sex, age, body weight, and nutritional status, can significantly alter the secretion pattern of GH, which can then in turn influence its biological effects.^9^, ^10^ Pit‐1, also called GHF‐1 or POU1F1, is a pituitary‐specific transcription factor of GH that plays a major role in both GH activation and pituitary development. In the human pituitary, *Pit‐1* gene mutation can lead to congenital GH deficiency (GHD).^11^ Studies from epidemiology and animal have shown that circulating GH levels are reduced in the context of HFD‐induced weight gain,^12^, ^13^ however, whether this process is caused by suppression of Pit‐induced GH synthesis needs to be explored.

The endoplasmic reticulum (ER) is a key intracellular organelle that plays an important role in regulating the synthesis of newly secreted proteins.^14^ ER homeostasis is mainly regulated by the unfolded protein response (UPR), which is a complex signaling system that regulates transcription and translation in order to increase the protein folding capacity of the ER.^15^ In some organs, such as the heart, overnutrition induced by HFD consumption can result in ER dysfunction, lead to ER stress, and activate the UPR.^16^ Three major pathways involved in the UPR are the inositol‐requiring enzyme 1α (IRE1α) signaling pathway, the protein kinase RNA‐like ER kinase (PERK) signaling pathway, and the activating transcription factor 6 (ATF6) signaling pathway.^17^ Interestingly, some recent studies have suggested that ER stress also exists in the central nervous system and contributes to neurodegenerative diseases.^15^, ^18^, ^19^

In this study, we hypothesized that lipotoxicity could inhibit the synthesis of GH in pituitary somatotrophs. To test this hypothesis, we first analyzed the association between serum TG and GH levels in a cross‐sectional population. Then, we fed rats either HFD or control diet (CD) and observed the levels of serum GH and the expression of proteins critical to GH synthesis and ER stress. We also used palmitic acid (PA)‐treated GH3 cells to study the direct effects of lipotoxicity on GH synthesis. Finally, rat and cell models of ER stress alleviation were generated by application of 4‐phenyl butyric acid (4‐PBA) or 4µ8c. Our results suggest that lipotoxicity can inhibit the synthesis of GH and that the IRE1α signaling pathway of ER stress may play an important role in this process.

## 2 MATERIALS AND METHODS

### 2.1 Subjects

We performed a cross‐sectional epidemiological investigation in Ningyang in Shandong Province, China, from June to November 2011. All of the participants were more than 40 years old and had lived there for more than 5 years. All participants provided written informed consent before data collection.

The exclusion criteria were as follows: females; individuals <40 or ≥60 years old; individuals suffering from diabetes mellitus, hypertension and hypothalamic or pituitary gland diseases; individuals suffering from complications or conditions that affect pituitary status and lipid metabolism such as malignant tumor and severe hepatic or renal dysfunction; individuals taking medicines that affect serum lipids in the previous three months; missing pivotal data such as age, sex, and serum lipid profiles; hypercholesterolemia. Then, we stratified the subjects according to age, fasting plasma glucose (FPG) levels, HbA1c levels, systolic blood pressure (SBP), and diastolic blood pressure (DBP). Ultimately, 90 men were included in our study.

### 2.2 Animals

All animal experiments were approved by the Animal Ethics Committee of Shandong Provincial Hospital. Male Sprague‐Dawley (SD) rats were purchased from Vital River Laboratory Animal Technology Co. Ltd. (Beijing, China) and fed in the experimental animal center of Shandong Provincial Hospital. The rats were kept under a 12:12 hours light:dark cycle at 22 ± 0.5°C and 50%‐60% humidity.

After 1 week of adaptive rearing, the rats were divided into two groups at random and fed either a CD (n = 15) (100% standard chow, 3.4 kcal/g) or an HFD (n = 15) (85% standard chow supplemented with 15% lard, 4.1 kcal/g). At the 0th, 4th, and 28th weeks of feeding, fasting blood samples were taken from the subclavian vein between 8:00 and 10:00 AM.

For GH stimulation test, at the end of feeding a CD or an HFD for 28 weeks, the rats were injected with 5 µg/kg GHRH via tail vein. The serum GH levels were tested by ELISA at 0, 8, 15, 20, and 30 minutes after GHRH administration.

For mechanistic studies, another 40 rats were randomly divided into four groups and fed a CD or HFD. From the 9th week, 4‐PBA (Sigma) was given daily at a dose of 100 mg/kg/d intraperitoneal injection (ip) to the rats in the CD+4‐PBA group and the HFD+4‐PBA group until the end of the 18th week. Correspondingly, equal volumes of PBS were given to the rats in the CD+PBS group and the HFD+PBS group at the same time, respectively.

### 2.3 Serum lipid parameter and GH/IGF‐1 hormone analyses

Serum TG levels were measured using enzymatic methods on an automatic biochemistry analyzer (Olympus, Japan). GH levels in serum and culture supernatant and serum IGF‐1 levels were measured using ELISA kits (CUSABIO, Wuhan, China). All steps were performed in strict accordance with the instructions.

### 2.4 Pituitary lipid concentration assays

Pituitary TG was extracted and assayed in strict accordance with the kit manufacturer's instructions (Applygen Technologies Inc). Pituitary‐free fatty acids (FFAs) were assayed using an FFA quantification kit (BioVision). The contents of TG and FFAs were normalized to those of the corresponding proteins.

### 2.5 Hematoxylin and eosin (H&E) and Oil Red O staining

Pituitary glands were fixed in 4% paraformaldehyde, dehydrated, embedded in paraffin, cut into 4‐μm thick sections. Consecutive sections in different fields in each pituitary were cut, and then at least four to five consecutive sections were collected and used in H&E staining. For Oil Red O staining of GH3, cells were fixed with 4% paraformaldehyde for 30 minutes before staining with Oil Red O solution (Goodbio Technology, Wuhan, China) according to the manufacturer's instructions.

### 2.6 Transmission electron microscopy (TEM) analysis

Pituitary glands were fixed in 3% glutaraldehyde for 2 hours and in 1% OsO_4_ for 1 hour. The fixed tissues were dehydrated in a graded series of ethanol washes and embedded in epoxy resin. Ultrathin sections were obtained and observed using a transmission electron microscope (H‐800, Hitachi).

### 2.7 Immunofluorescence

A 4% paraformaldehyde‐fixed GH3 cells were incubated with goat anti‐GH antibodies (1:200, sc‐10365, Santa Cruz) and then with FITC‐conjugated IgG (1:1000, Thermo Fisher). For Pit‐1 staining, cells were incubated with rabbit anti‐Pit‐1 antibodies (1:200, sc‐442, Santa Cruz) followed by TRITC‐conjugated IgG (1:1000, Invitrogen). The nuclei were stained with DAPI (1:1000, Invitrogen). After treatment, the cells were immediately observed under a fluorescence microscope.

### 2.8 RNA isolation and real‐time quantitative qPCR

Total RNA from the whole pituitary glands and GH3 cells was isolated and real‐time quantitative RT‐PCR was used to determine relative mRNA expression of rat *Gh*, *Pit‐1,* and *β‐actin*. We carried out in accordance with a method previously described.^6^

### 2.9 Protein extraction and Western blot

For total protein extraction and Western blot, we carried out in accordance with a method previously described.^20^ The blots were probed with the following primary antibodies: BiP (1:1000, Proteintech, 11587‐1‐ap), p‐IRE1α (1:1000, Abcam, ab124945), IRE1α (1:1000, Abcam, ab37073), p‐eIF2α (1:1000, CST, 3597), eIF2α (1:1000, CST, 9722), ATF6 (1:1000, Abcam, ab122897), XBP1 (1:1000, sc‐7160, Santa Cruz), Pit‐1 (1:1000, sc‐442, Santa Cruz), GH (1:1000, sc‐10365, Santa Cruz), and GAPDH (1:7500, 60004‐I‐Ig, Proteintech). The blots were incubated with corresponding secondary antibodies conjugated to horseradish peroxidase (HRP) (ZSGB‐ Bio) at a dilution of 1:5000.

### 2.10 GH3 cell culture and treatment

GH3 cells were maintained in low‐glucose DMEM (HyClone) supplemented with 10% FBS (Gibco). Cells grown to approximately 60%‐80% confluence were moved to starvation medium supplemented with 2% FBS. Two hours later, the cells were treated with PA (Sigma) diluted in BSA (Sigma) solution at the indicated concentration and time, and the corresponding control group was treated with the same amount of BSA alone. To study the effect of ER stress, 5 mmol/L 4‐PBA (an inhibitor of ER stress; Sigma) or 30 μmol/L 4μ8c (an inhibitor of IRE1α; Millipore) was used 1 hour prior to PA treatment for the indicated time.

### 2.11 Statistical analysis

The values are expressed as the mean ± standard deviation (SD). All data were analyzed using SPSS version 18.0. The relationship between serum TG and GH levels was assessed through Spearman correlation analysis and multivariate linear regression. Means were compared using Student's *t*‐test for comparisons between two groups and one‐way ANOVA for comparisons among multiple groups. *P* < .05 was considered to indicate significance.

## 3 RESULTS

### 3.1 Epidemiological analysis showed that serum GH levels were correlated negatively with TG levels in the population

**FIGURE 1:**
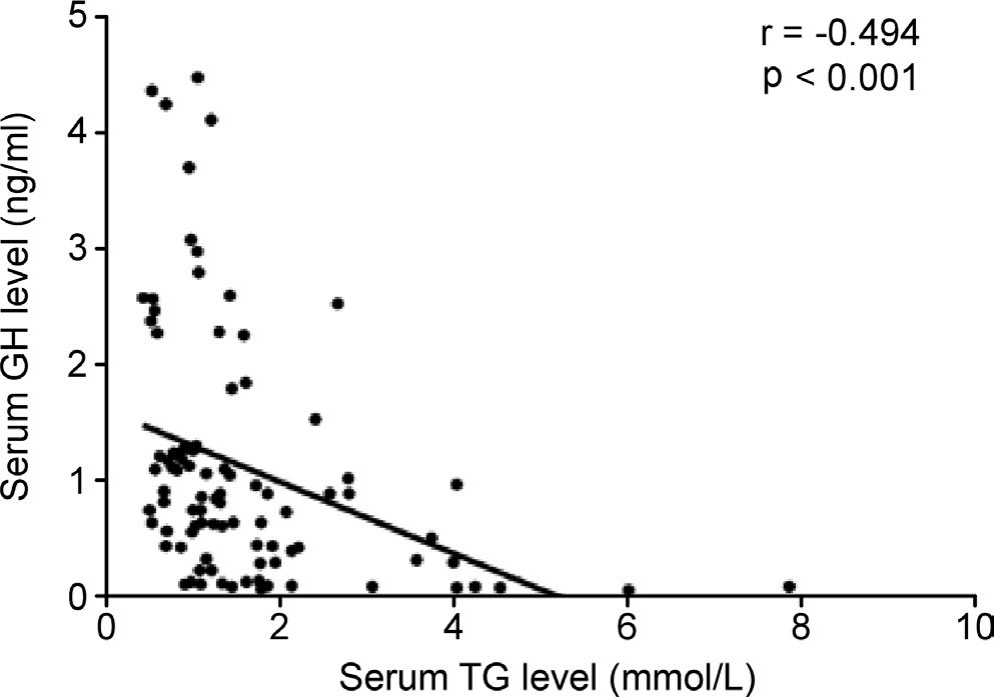
Growth hormone (GH) was correlated negatively with serum TG level in the human population. Scatter diagram of serum GH and triglyceride (TG) levels in humans. The correlation coefficient (r) and significance (*P*) are marked in the figure

To determine the correlation between serum levels of GH and TG, we carried out a cross‐sectional population study. Considering that serum pituitary hormone levels change greatly during the female menstrual cycle, we only include male as our subjects. After exclusion of subjects with conditions that may affect pituitary function and serum lipid profiles, 90 volunteers were enrolled in our study. As shown in Figure 1, among all volunteers, GH levels were negatively correlated with TG levels (*r* = −.494, *P* < .001). Then, we performed multiple linear regression to exclude the influences of known confounding factors. Interestingly, the correlations remained significant (β = −0.279, *P* = .009) after adjustment for age, BMI, FPG level, and SBP. Together, these results provided important insights into the negative correlation between TG levels and GH levels; however, there was no direct evidence that TG decreased GH levels.

### 3.2 Effect of the HFD on serum TG levels and the GH/IGF‐1 axis in rats

**FIGURE 2:**
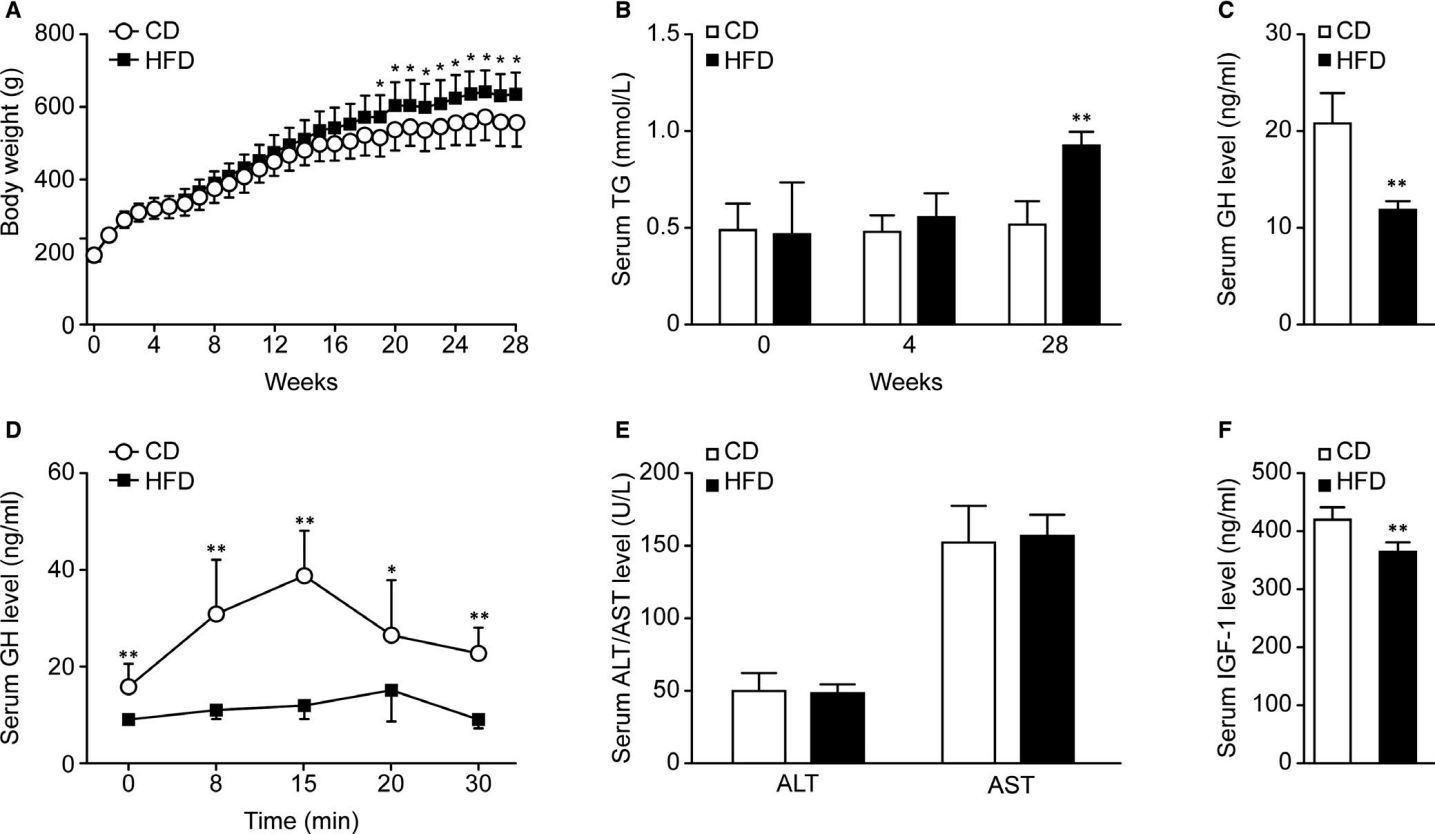
Effect of HFD on body weight, serum TG levels, and the GH/IGF‐1 axis in rats. Rats were fed a CD or an HFD for 28 wk. A, The body weights of the rats were monitored weekly (n = 10). B, The serum TG levels were detected at the 0th, 4th, and 28th weeks (n = 6). C, Basal serum GH levels at the 28th week (n = 5). D, Serum GH concentrations in rats in response to intravenous injection of GHRH (n = 4‐7). E, The serum ALT and AST levels at the 28th week (n = 6). F, Serum IGF‐1 levels at the 28th week (n = 5). *P < .05; **P < .01

The results above show that serum GH levels were negatively correlated with serum TG levels. To determine whether lipotoxic environment decreased serum GH levels, rat model with hypertriglyceridemia induced by an HFD was established. The body weights of the rats were monitored weekly during the entire 28‐week feeding period (Figure 2A). The rats in the HFD group showed higher body weights than those in the CD group. To assess whether we successfully established hypertriglyceridemia model, we examined the serum levels of TG at the end of 0, 4, and 28 weeks, respectively (Figure 2B). There was no significant difference in serum TG levels between the two groups at the 4th week. However, at the 28th week, the rats in the HFD group showed significantly higher serum TG levels than those in the CD group (*P* < .01).

After determining whether HFD consumption could induce hypertriglyceridemia in rats, we observed whether serum GH levels were altered following HFD feeding. We measured the basal serum levels of GH at the 28th week. Figure 2C shows that the serum GH levels were obviously lower in the HFD group than in the CD group (*P* < .01). To evaluate the function of somatotroph cells in the pituitary, animals fed the CD or HFD for 28 weeks were subjected to a GH stimulation test. In our study, we chose GHRH as the single secretagogue and stimulated GH peaks in the rats in both the CD and HFD groups (Figure 2D). The figure shows that the GH peak of the HFD group appeared at 20 minutes, later than that of the CD group, which appeared at 15 minutes. Mean peak GH levels were significantly lower in the HFD group than in the CD group. This finding strongly demonstrates that HFD consumption reduced the reserve function of somatotroph cells. The main function of peripheral GH is to regulate the synthesis of IGF‐1 in the liver. Under the premise that the rat liver function remains unchanged (Figure 2E), Figure 2F shows a significant decrease in serum IGF‐1 levels in the HFD group at the 28th week. This finding further confirmed the reductions in GH levels in the circulation.

### 3.3 Lipotoxicity induced a decrease in GH expression with disturbance of anterior pituitary morphology

**FIGURE 3:**
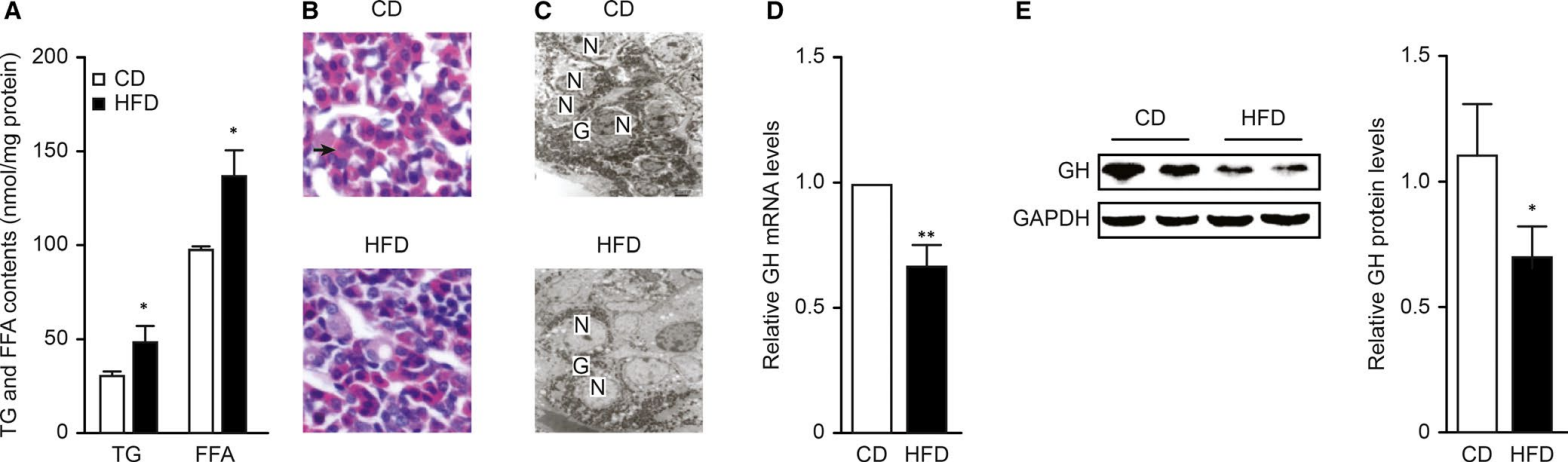
Lipotoxicity induced a decrease in GH expression with disturbance of anterior pituitary morphology. Rats were fed a CD or an HFD for 28 wk. A, Analysis of TG and FFA levels in rat pituitary tissue; the concentrations were normalized to the corresponding protein levels (n = 4). B, Histological changes in the anterior pituitary as indicated by H&E staining (magnification, 200×). Acidophilic cells are noted by green arrows. C, Representative electron micrographs of anterior pituitaries from CD and HFD rats. N, nucleus; G, secretory granule. Scale bar = 2μm. D, mRNA levels of GH in the whole anterior pituitaries of CD and HFD group rats (n = 5). E, Western blot analysis of GH protein levels in the whole anterior pituitaries of CD and HFD groups (n = 6). *P < .05; **P < .01

The pituitary is more easily affected by blood lipid levels than other parts of the brain because it has no blood‐brain barrier. To determine the effect of HFD consumption on lipid content in the pituitary gland, we measured both TG and FFA levels of pituitary tissues at the 28th week. Figure 3A shows the TG and FFA levels in rat pituitary tissue. Both TG and FFA levels were significantly higher in the HFD group (both *P* < .05) than in the CD group, indicating excess lipid accumulation in pituitary tissues, which might be a prerequisite for lipotoxicity.

The somatotroph cells in the pituitary gland are acidophilic cells. The histomorphometry findings (Figure 3B) showed that pituitary tissue from the CD group showed normal features under light microscopy, and acidophilic cells were observed. However, acidophilic cells in the HFD group were significantly reduced. The electron micrographs (Figure 3C) showed that in the CD group, normal numbers of secretory cells were present in the pituitary gland and were surrounded by abundant secretory granules. However, in the HFD group, the numbers of both secretory cells and granules were decreased.

The serological data did not fully reflect the reserve function of GH in the pituitary gland. To survey whether HFD consumption was connected with the protein expression of GH in the pituitary gland, we directly evaluated the mRNA and protein levels of GH in pituitary tissue (Figure 3D,E). The qPCR and Western blot results revealed that both the mRNA and protein levels of GH were reduced in pituitary tissue after 28 weeks of HFD feeding.

### 3.4 PA directly suppressed the synthesis of GH in GH3 cells

There are many confounding factors of HFD‐induced hypertriglyceridemia in animal models, including weight gain, body composition, and the levels of hormones such as leptin, insulin, and ghrelin.^21^ PA is the most abundant saturated fatty acid in the serum of HFD‐fed rats,^22^ and GH3 is a well‐characterized pituitary cell line that synthesizes and secretes GH.^23^ We treated GH3 cells with PA to survey whether lipotoxicity could directly suppress the synthesis of GH.

**FIGURE 4:**
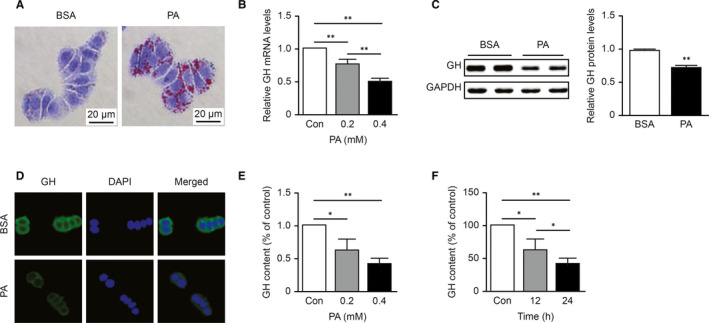
Palmitic acid directly suppressed the synthesis and secretion of GH in GH3 cells. A, Oil Red O staining of GH3 cells treated with BSA or PA (0.2 mmol/L) for 24 h. B, The dose‐dependent effect of PA on the mRNA level of GH in GH3 cells was detected by qPCR (n = 3). C, Western blot analysis of GH protein levels in PA (0.2 mmol/L, 24 h)‐treated GH3 cells (n = 5). D, The cellular distribution of GH (shown in green) after PA treatment (0.2 mmol/L, 24 h) was visualized by immunofluorescence in GH3 cells. The nuclei were stained with DAPI (shown in blue). E,F, Dose‐dependent effect of PA (24 h) and time‐dependent effect of PA (0.2 mmol/L) on GH content in culture supernatant (n = 3).*P < .05; **P < .01

As shown in Figure 4A, Oil Red O staining revealed significant increases in lipid levels in GH3 cells after they were treated with PA (0.2 mmol/L) for 24 hours, suggesting that GH3 cells are also susceptible to lipotoxicity. Consistent with the findings of the animal experiments, PA reduced GH mRNA and protein expression in GH3 cells (Figure 4B,C). Immunofluorescence also demonstrated the inhibitory effect of PA on GH protein expression (Figure 4D). As a consequence, the extracellular GH concentrations decrease in a dose‐ and time‐dependent manners (Figure 4E,F). Together, these results showed that both intra‐ and extracellular GH levels were reduced, indicating that PA could directly suppress the synthesis of GH in GH3 cells.

### 3.5 Lipid overload reduced the expression of Pit‐1 in GH3 cells and the pituitary

**FIGURE 5:**
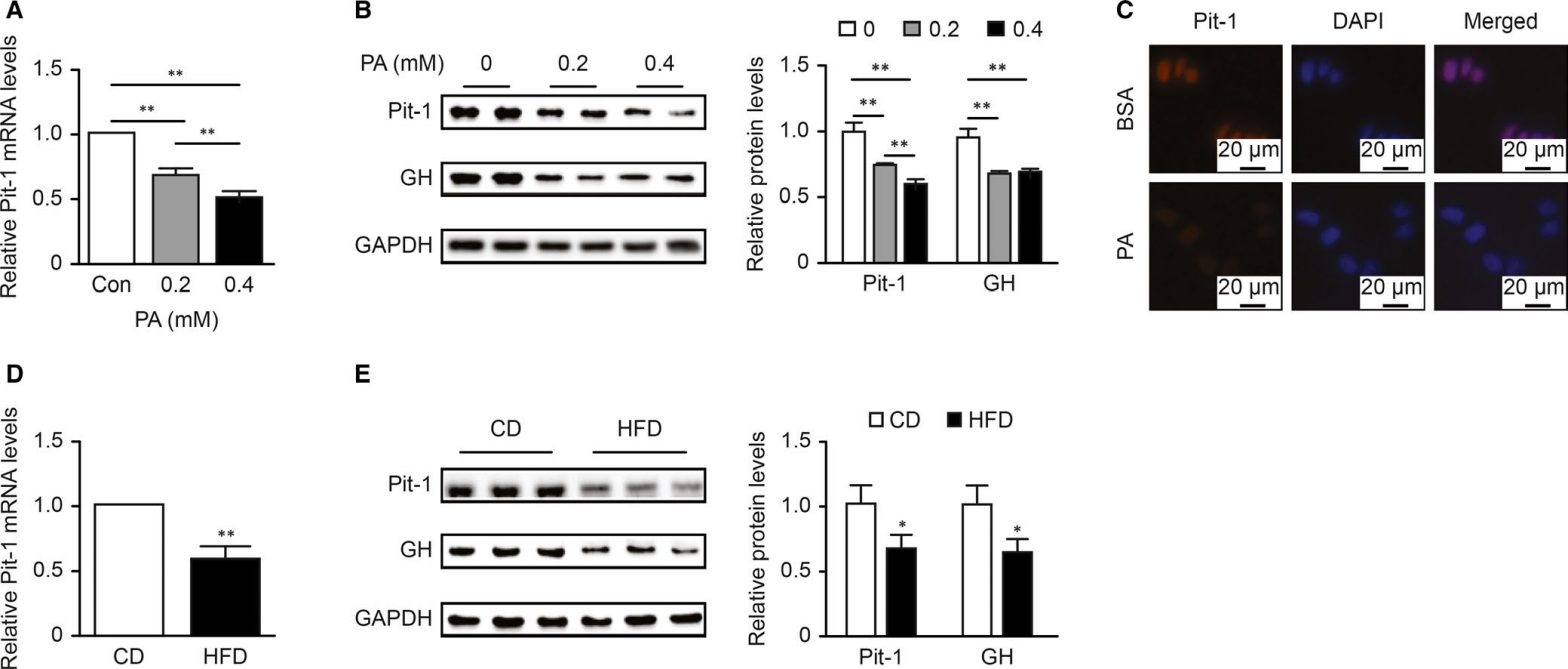
Lipid overload reduced the expression of Pit‐1 in GH3 cells and pituitaries. A, The dose‐dependent effect of PA at 24 h on Pit‐1 mRNA levels in GH3 cells was detected by qPCR (n = 3). B, The dose‐dependent effects of PA at 24 h on Pit‐1 and GH protein levels in GH3 cells were detected by Western blot (n = 4). C, Immunofluorescence staining of Pit‐1 after PA treatment (0.2 mmol/L, 24 h) in GH3 cells. D, qPCR analysis of Pit‐1 mRNA levels in the whole anterior pituitaries of CD and HFD groups (n = 5). E, Western blot analysis of Pit‐1 and GH protein levels in the whole anterior pituitaries of CD and HFD groups (n = 6). The values were quantified by densitometry and normalized to GAPDH values. *P < .05; **P < .01

As described above, Pit‐1 is a major transcription factor that regulates GH synthesis. To examine the influence of lipotoxicity on Pit‐1 in vivo and in vitro, we detected the expression of Pit‐1 by qPCR, Western blot and immunofluorescence. First, as shown in Figure 5A,B, PA reduced Pit‐1 mRNA and protein expression in GH3 cells in a dose‐dependent manner. Moreover, as expected, the fluorescence intensity of Pit‐1 was weak in the PA group compared with the control group (Figure 5C). Then, we examined whether this phenomenon existed in vivo. Figure 5D,E show that compared with that in the CD group, the expression of Pit‐1 mRNA and protein in pituitary tissue in the HFD group was significantly reduced. Taken together, these results indicated that lipotoxicity could reduce the expression of Pit, a key transcription factor for GH, which might have led to a decrease in GH synthesis in the rats.

### 3.6 Lipotoxicity triggered ER stress in pituitary somatotrophs, and blocking ER stress or the IRE1α signaling pathway attenuated the decreases in Pit‐1 and GH

**FIGURE 6:**
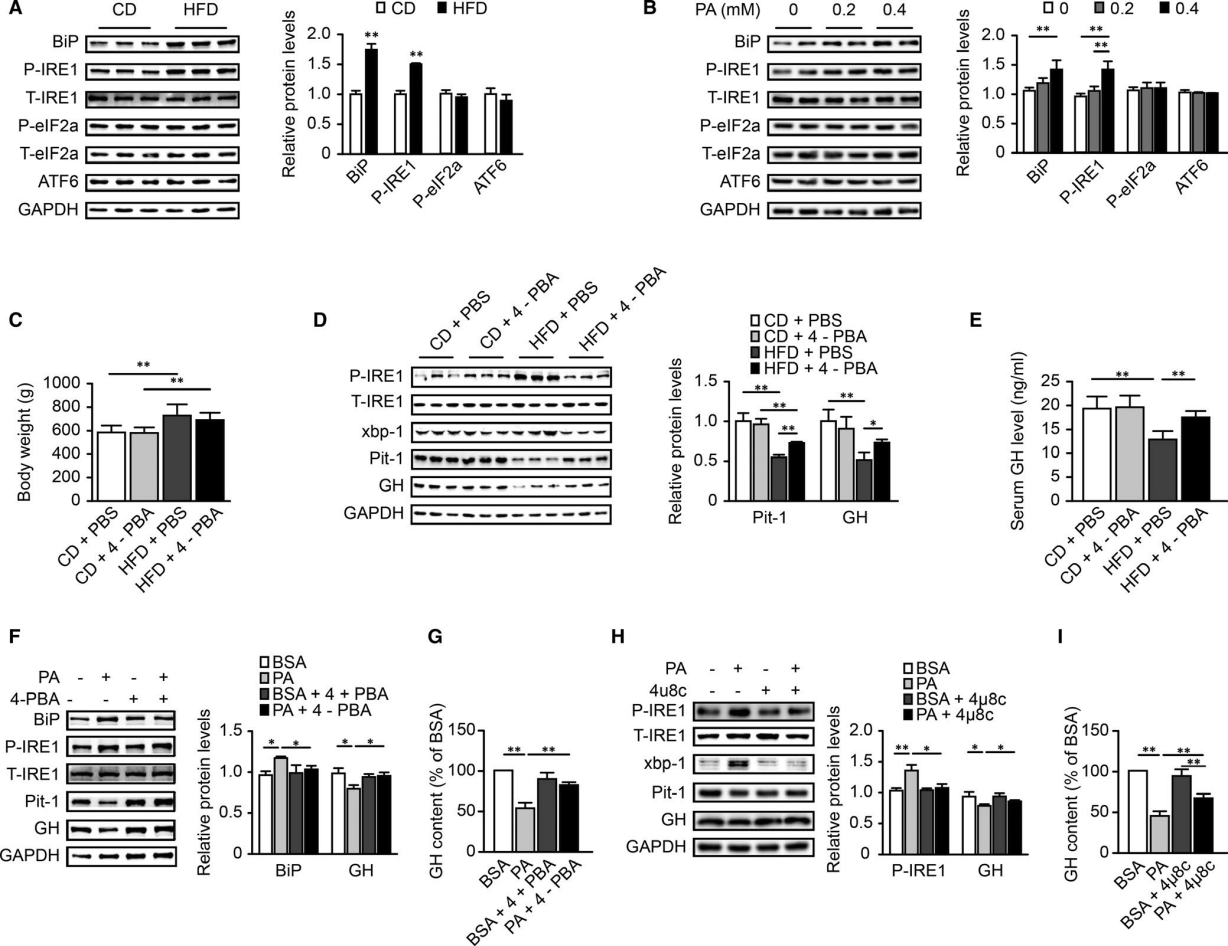
Lipotoxicity triggered ER stress in pituitary somatotrophs, and blocking ER stress or the IRE1α signaling pathway attenuated the decreases in Pit‐1 and GH. A, Western blot analysis of BiP, IRE1α, eIF2α, and ATF6 activation levels in the CD and HFD groups (n = 6). The values were quantified by densitometry. The phosphorylated IRE1α and eIF2α levels were normalized to the respective total protein levels, while the BiP and ATF6 levels were normalized to the GAPDH levels. B, Western blot analysis of BiP, IRE1α, eIF2α, and ATF6 activation levels in GH3 cells treated with PA (0.2 mmol/L or 0.4 mmol/L, 24 h) by Western blot (n = 4). (C‐E), The rats in the CD+4‐PBA and HFD+4‐PBA groups were given 4‐PBA (100 mg/kg/d ip) from the 9th week to the 18th week. The vehicle, PBS, was given correspondingly as a control in the CD+PBS and HFD+PBS groups. C, Body weights of rats (n = 11‐16). D, Western blot analysis of Pit‐1 and GH protein levels and IRE1α and XBP‐1 activation levels (n = 5‐6). The values were quantified by densitometry, and the Pit‐1 and GH levels were normalized to the GAPDH levels. E, Basal GH level of each group (n = 5‐8) (F,G) GH3 cells were cultured with 5 mmol/L 4‐PBA 1 h prior to treatment with PA (0.2 mmol/L, 24 h). F, Western blot analysis of Pit‐1 and GH protein levels and BiP and IRE1α activation levels (n = 3). G, GH content in culture supernatant (n = 3). (H,I) GH3 cells were cultured with 30 μmol/L 4μ8c (inhibitor of IRE1α) 1 h prior to treatment with PA (0.2 mmol/L, 24 h). H, Western blot analysis of Pit‐1 and GH protein levels and IRE1α and XBP‐1 activation levels (n = 3). I, GH content in culture supernatant (n = 3). *P < .05; **P < .01

Increased FFA levels, particularly saturated fatty acid levels, have been linked to ER stress activation in a number of cell types.^24^ To study whether lipotoxicity causes ER stress in pituitary somatotrophs, we analyzed ER stress indicators by Western blot. As shown in Figure 6A, the expression of BiP and the phosphorylation of IRE1α in pituitary tissue were significantly greater in the HFD group than in the CD group, while the expression of eIF2α‐ and ATF6‐related pathway members was not significantly increased. Then, we tested the results in vitro with GH3 cells treated with PA. Consistent with the in vivo results, PA treatment induced ER stress in GH3 cells, activating BiP and IRE1α (Figure 6B). These results indicated that ER stress and the IRE1α signaling pathway were induced in the pituitary somatotrophs of HFD rats. This change might have been one of the factors causing dysfunction of GH synthesis.

To further illustrate the role of ER stress in lipotoxicity‐induced pituitary somatotroph dysfunction and to explore possible treatment strategies, 4‐PBA, which is a chemical chaperone inhibiting ER stress, was administered to the rats by intraperitoneal injection. Serum GH levels and GH biosynthesis function in the pituitary were monitored. As shown in Figures 6D, 4‐PBA alleviated the activation of IRE1α and X‐box binding protein 1 (XBP‐1) in the HFD+4‐PBA group, compared with the HFD+PBS group, reflecting alleviation of ER stress under 4‐PBA treatment. In addition, the protein levels of Pit‐1 and GH in HFD+4‐PBA rats were higher than those in HFD+PBS rats. The HFD+4‐PBA rats also showed higher serum GH levels than the HFD+PBS rats (Figure 6E), suggesting the improvement of GH synthesis function under 4‐PBA treatment.

In vitro, we treated GH3 cells with 4‐PBA and 4µ8c (an inhibitor of IRE1α) to explore the roles of ER stress and the IRE1α signaling pathway. As expected, after treatment of 4‐PBA, the activation of BiP and IRE1α in GH3 cells was significantly reduced. GH levels in GH3 cells and culture supernatants were higher in the PA+4‐PBA group than in the PA group (Figure 6F,G). The effect of 4µ8c on GH synthesis was the same (Figure 6H,I). These results suggested that ER stress and IRE1α signaling pathways played important roles in the regulation of GH synthesis.

## 4 DISCUSSION

In the present study, we demonstrated that serum GH levels were negatively correlated with TG levels in an epidemiological study. We also observed that lipotoxicity could inhibit the synthesis of GH in vivo and in vitro, with decreased Pit‐1 expression. The IRE1α signaling pathway of ER stress was activated in HFD‐fed rats and PA‐treated GH3 cells. In addition, when we used 4‐PBA to alleviate ER stress in HFD rats, the decreases in Pit‐1 and GH were ameliorated. The same phenomenon also occurred in GH3 cells treated with 4‐PBA or 4µ8c. Our new findings provide a unique approach to manage GHD in hypertriglyceridemia.

Our epidemiological analysis revealed that GH was correlated negatively with serum TG in the population. This phenomenon has also been observed in clinical practice. In a study on children, Stawerska et al^25^ found significantly higher concentrations of TG in patients with GHD than that in controls, whereas they found no difference between the groups with regard to the concentrations of cholesterol and its fractions. However, the causality of this correlation needs to be explored in depth. Ohara E et al^26^ also reported an interesting clinical study in which decreased GH secretion was correlated strongly and negatively with BMI, and GH secretion in response to a GHRP‐2 load was improved after weight loss post laparoscopic sleeve gastrectomy. These data show that GH is very sensitive to nutritional statuses such as lipid overload.

As mentioned earlier, the deleterious effects of lipid accumulation in nonadipose tissues are collectively known as lipotoxicity. Previous studies have shown that lipotoxicity can cause dysfunction of many kinds of cells and tissues such as liver, heart, skeletal muscle, and lung tissues.^27^, ^28^, ^29^, ^30^ For example, in human liver cells, lipotoxicity can induce apoptosis via the PERK/ATF4/CHOP signaling pathway, which might be one of the pathogeneses of nonalcoholic fatty liver.^27^ The results of this study showed that both TG and FFA levels in the pituitary gland were significantly higher in the HFD group (both *P* < .05) than in the CD group. Similar results were obtained in our previous study.^6^ We also observed reduced GH synthesis in the anterior pituitary. These results reveal that lipotoxicity can affect pituitary somatotrophs. Morphology scanning revealed that the numbers of both secretory cells and granules decreased after HFD feeding in rats. Renier et al^31^ also reported that in Zucker rats with genetically hypertriglyceridemia, the total numbers of somatotroph cells are reduced. However, according to their study, Zucker rats have normal GH levels per somatotroph cell. This finding is different from that of our research; we treated GH3 cells with PA for 24 hours in vitro and found that GH content was obviously reduced. We think this difference may be caused by the different genetic background of hypertriglyceridemia rats.

Endoplasmic reticulum stress is a kind of adaptive response to the accumulation of misfolded proteins in the lumen of the ER. Moderate ER stress plays a protective role by activating the UPR, but excessive and long‐term ER stress leads to apoptosis.^32^ Numerous studies have intensely investigated the links between ER stress and obesity‐related diseases. In recent years, some studies have suggested that ER stress also exists in the central nervous system and contributes to neurodegenerative diseases. Cai et al^33^ reported that hippocampal ER stress affects the levels of neuronal plasticity‐related proteins (like BDNF) in rats with HFD‐induced obesity. In addition, HFD feeding can impair insulin receptor signaling in the hippocampus and frontal cortex by activating the PERK and IRE1α pathways of ER stress.^34^ Furthermore, in 2019, our team revealed that ER stress decreased thyroglobulin and contributed to HFD‐induced hypothyroidism.^20^ Taken together, these findings indicate that ER stress is vital in both central nervous system regulation and hormone homeostasis. However, no study has explored whether HFD consumption or lipotoxicity causes ER stress in the pituitary. In our research, the IRE1α signaling pathway was activated in the pituitary by HFD consumption or by elevated PA in vitro; however, the expression of ATF6α and the phosphorylation of eIF2α did not show obvious changes. Emerging evidence has revealed that IRE1α is a major signal transducer that responds to metabolic cues and nutrient stress conditions.^35^ We confirmed the lipid‐sensing effect of IRE1α in the pituitary gland. Our study reveals, for the first time, that lipotoxicity activates the IRE1α signaling pathway of ER stress in pituitary tissue, which might play a key role in the endocrine status disruptions, such as GHD, that occur in diet‐induced obesity.

We observed the occurrence of ER stress in both HFD‐fed rats and PA‐treated GH3 cells, accompanied by the activation of the IRE1α signaling pathway. The ER stress inhibitor 4‐PBA could improve GH reduction caused by lipotoxicity both in vivo and in vitro. Therefore, we were convinced that ER stress plays an important role in lipotoxic‐induced impairment of GH synthesis. Indeed, induction of ER stress is supposed to induce all the three UPR sensors without exceptions. However, depending on the observation time, there may be some differences in the activated pathways. Only the activation of the IRE1α pathway was observed in our specific setup, which did not mean that the PERK and ATF6 pathways did not work, but that these two pathways happened to be turned off during the time we observed. More recently, literature has shown that the IRE1α can respond directly to lipotoxicity through various means: (a) Under the environment of lipotoxic‐induced ER stress, IRE1α branch was activated by detecting unfolded proteins and chaperones via the IRE1α luminal domain; (b)Under the condition of lipid metabolism disorder, IRE1α‐sensed membrane lipid saturation via its transmembrane domain and was activated directly.^36^, ^37^, ^38^, ^39^ In our study, whether the activation of the IRE1α pathway is secondary to the UPR/ER stress or membrane lipid saturation needs to be explored.

Under physiological conditions, IRE1α is associated with BiP and remains in a repressed state. When ER stress occurs, BiP dissociation leads to IRE1 activation by dimerization and trans‐autophosphorylation. Activated IRE1α possesses endoribonuclease (endoRNase) activity at the carboxyl end of its cytoplasmic domain and splices 26 nucleotides from the mRNA encoding XBP‐1. Spliced XBP‐1 translocates into the nucleus to promote transcriptional programs that up‐regulate a broad spectrum of UPR‐associated genes.^40^ Long‐term ER stress augments the endoRNase activity of IRE1α, thereby leading to decreased specificity for XBP‐1 mRNA and elevated degradation of other classes of mRNAs through regulated IRE1‐dependent decay (RIDD).^41^ However, the specific molecular mechanism is still unclear. In our study, lipotoxicity reduced the mRNA levels of *Pit‐1* and *Gh*, and these reductions were accompanied by activation of the IRE1α signaling pathway. Whether the reduced mRNA expression of *Pit‐1* and *Gh* was caused by RIDD requires further exploration.

This study has a potential limitation. For the epidemiological analysis, we used a cross‐sectional study to analyze the relationship between serum TG levels and GH levels. The results were not able to indicate whether there was a causal relationship between these parameters. Our experiments on rats and in vitro made up for this limitation.

In conclusion, our findings revealed that GH levels were correlated negatively with serum TG levels in the study population. Lipotoxicity directly suppressed the expression of Pit‐1 and the synthesis of GH, but these effects were attenuated by blockade of the IRE1α pathway of ER stress. This study provides new insight into the effects of lipotoxicity on pituitary somatotroph function and these may be therapeutic targets for GHD in obesity.

## CONFLICT OF INTEREST

All the authors declare no conflicts of interest.

## AUTHOR CONTRIBUTIONS

**Ying Gong:** Data curation (lead); Formal analysis (lead); Investigation (lead). **Jianmei Yang:** Data curation (supporting); Formal analysis (supporting); Investigation (supporting). **Shuoshuo Wei:** Data curation (supporting); Investigation (supporting). **Rui Yang:** Data curation (supporting); Investigation (supporting). **Ling Gao:** Funding acquisition (supporting); Project administration (supporting). **Shanshan Shao:** Funding acquisition (supporting); Project administration (lead). **Jiajun Zhao:** Funding acquisition (lead); Project administration (lead).
